# Biotechnological and Medical Aspects of Lactic Acid Bacteria Used for Plant Protection: A Comprehensive Review

**DOI:** 10.3390/biotech11030040

**Published:** 2022-08-31

**Authors:** Simon Bergsma, Gerrit Jan Willem Euverink, Nikolaos Charalampogiannis, Efthymios Poulios, Thierry K. S. Janssens, Spyridon Achinas

**Affiliations:** 1 Faculty of Science and Engineering; University of Groningen, Nijenborgh 4, 9747 AG Groningen, The Netherlands; 2 SLK Kliniken am Gesundbrunnen, Am Gesundbrunnen 20–26, 74078 Heilbronn, Germany; 3 4th Department of Surgery, Attikon University Hospital, Medical School, National and Kapodistrian University of Athens, Rimini 1, Chaidari, 12462 Athens, Greece; 4 Biotech Microbials BV, Distributieweg 1, 2645 EG Delfgauw, The Netherlands

**Keywords:** pesticides, LAB, fermentation, agriculture, human health

## Abstract

The use of chemical pesticides in agriculture goes hand in hand with some crucial problems. These problems include environmental deterioration and human health complications. To eliminate the problems accompanying chemical pesticides, biological alternatives should be considered. These developments spark interest in many environmental fields, including agriculture. In this review, antifungal compounds produced by lactic acid bacteria (LABs) are considered. It summarizes the worldwide distribution of pesticides and the effect of pesticides on human health and goes into detail about LAB species, their growth, fermentation, and their antifungal compounds. Additionally, interactions between LABs with mycotoxins and plants are discussed.

## 1. Introduction

Plants are susceptible to fungal infections. Fungi deteriorate crops so that they are no longer viable as food for people. Worldwide, the total amount of food wasted due to fungal growth and production of toxic compounds is priced at billions of dollars [1]. During the colonization of plants, fungi also produce toxic compounds, the so-called mycotoxins. These compounds can be harmful to animals, as well as humans [1,2]. To protect the plants against these successful invaders, thereby increasing yields and maximizing gains, research efforts to develop biocontrol agents have to be performed.

The green revolution in the 1970s provided farmers with pesticides [3]. Fungicides, pesticides that specifically target fungi, are substances that inhibit fungal growth (fungistatic) or completely kill the fungus (fungicidal). The downsides of using these fungicides include contamination of the soil, negative effects on human health, and the development of resistance of the pathogens to the used fungicides [4,5].

Lactic acid bacteria (LABs) are Gram-positive, low-GC, aerotolerant, rod- and coccus-shaped, non-spore-forming organisms which lack catalase and a respiratory chain [6]. LABs are classified as phylum Bacillota (synonym Firmicutes), class Bacilli, and order Lactobacillales. They are known, as their name suggests, to produce lactic acid. This is the main product after fermentation of the carbon source. LABs can ferment carbohydrates via homo fermentation and hetero fermentation.

LABs are used in a variety of fermentation processes [7,8] The function of LABs in these processes can vary from enhancing the taste of the products to acting as a preservative [9]. LABs can preserve food with the production of acids in fermentation. These bacteria enjoy the generally regarded as safe (GRAS) status in the US and the qualified presumption of safety (QPS) status in the EU [6,10,11,12]. This makes these organisms economically interesting for investigation and utilization.

The main goal of this study was to review trials of using LABs to protect crops against fungi. As described earlier, the use of chemical fungicides has a lot of serious disadvantages. Additionally, with the world becoming more aware of sustainable agriculture and organic farming, interest in this topic will also increase.

## 2. Current Status of Pesticides

The first recorded use of pesticides is from the ancient Sumerians; they used elemental sulfur (S) and chemicals containing arsenic (As) and lead (Pb) [13]. During the Second World War, the production of synthetic weapons automatically steered toward the production of chemical pesticides, with the most notable one being the pesticide DDT (dichlorodiphenyltrichloroethane). This pesticide played an intrinsic role in the fight against malaria. Unfortunately, negative effects on other animals, such as birds and fish, were discovered. For humans, DDT is also toxic and can induce the development of different kinds of cancer, most notably breast cancer.

Nowadays, the total use of pesticides still increases each year [14]. Pesticides increase the yield of crops drastically. Pesticides are, therefore, necessary to sustain the growing world population. It will be interesting to look at the spread of the use of pesticides around the world and the development of the use of pesticides in each part of the world (Table 1).

Approximately 10% of all pesticides are fungicides or bactericides. Between 1990 and 2014, their use increased by around 50% [14]. The increase in fungicide use demands an eco-friendlier alternative to not increase the environmental impact of fungicides. Pests also can develop resistance; alternative fungicides can be a great way to avoid fungal resistance to traditional fungicides.

## 3. Transition to Biopesticides: A Human Health-Based Perspective

As described in the previous section, pesticides can also affect human health. Pesticides can affect occupational groups (i.e., people who work in agriculture), the general population that lives in highly polluted areas, and consumers of food that has been treated with pesticides, as residues of those pesticides can remain in the food until it is consumed [16,17,18]. Within the body, pesticides can be metabolized, excreted, and stored in body fat [19,20].

### Negative Effects of Fungicides Compared to Biofungicides

The major classes of fungicides are triazoles, phenylpyrroles, strobilurins, benzimidazoles, and morpholines [21]. Researchers looked at the effect of the chiral triazole prothioconazole and its metabolites, which are also EDCs [22]. Using molecular docking, they found that, while prothioconazole bound well to the receptors TRb (thyroid hormone receptor) and Erα (estrogen receptor), its metabolites could bind even better. By binding to these receptors, the EDCs can inhibit or disrupt the synthesis, secretion, and metabolism of hormones. This can affect the developmental and reproductive systems in humans [22]. Just like some insecticides and herbicides, fungicides can have negative health effects on the thyroid. In this study, the authors checked the effect of several pesticides on human health. Shresta et al., (2018) tested female spouses of farmers working with pesticides, as women are showing a higher susceptibility to pesticides regarding thyroid abnormalities [23]. The authors note that although results are inconclusive, the fungicide maneb appears to change the concentration of thyroid-stimulating hormone (TSH), which alters the function of the thyroid. To describe the effects of pesticides on the likeliness of people to get a disease, the researchers use a hazard ratio (HR). An increase of 50% in the likelihood to catch a disease corresponds with an HR of 1.5. The HR for the fungicide benomyl was 1.21, and for the fungicide maneb/mancozeb, this value was 1.44. This means that people that were exposed to these fungicides had a significantly higher chance to develop hypothyroidism. Copper-based fungicides also inhibit the growth of certain bacteria, for example, nitrogen-fixating bacteria, that the plant requires for root growth [24]. Interactions between plants and their microbiome can enhance the production of secondary metabolites produced by the plant [25]. The addition of fungicides can, by disturbing these interactions, affect the texture, taste, and nutritional value of vegetables. The use of bacteria as biocontrol agents is receiving more and more attention, as some strains can induce systemic resistance of plants against pathogens [26]. This would reduce the need for chemical pesticides. Human health would also benefit from unprocessed organic food, as it supplies more health-promoting microbes and secondary metabolites produced by both plants and bacteria [25]. When comparing biopesticides (including fungicides) to pesticides, researchers say that the toxicological risk of biopesticides is lower than the risks of regular pesticides [27]. This is caused by the higher specificity of biopesticides, meaning that they have less or no effect on organisms that are not targeted by them. Apart from the benefits for human health, soil contamination and water contamination will also be reduced when a lower amount of chemical pesticides, such as organophosphate and carbamate pesticides, is used [28]. Especially in developing countries, the residual presence of pesticides in drinking water is very troubling.

## 4. LAB Species and Their Antifungal Compounds

### 4.1. Antifungal Compounds Produced by LABs

The great variety of LAB species is crucial in the fight against the different fungi, as different LAB species will produce different secondary metabolites. In Table 2, different groups and different strains producing different secondary metabolites, as well as their antifungal properties, are given. These antifungal properties are caused by different antifungal agents and have been examined against different strains of fungi. LAB species can be both heterofermenters and homofermenters. This means that they can either produce one kind (homofermenters) or several kinds of organic acids (heterofermenters). When one kind of acid is produced, this usually means that the total yield of this organic acid is higher [29]. When several acids are produced, the total yield of acids is usually lower, but several acids might have synergetic effects in regard to the inhibition of fungal growth [6].

**Figure 1:**
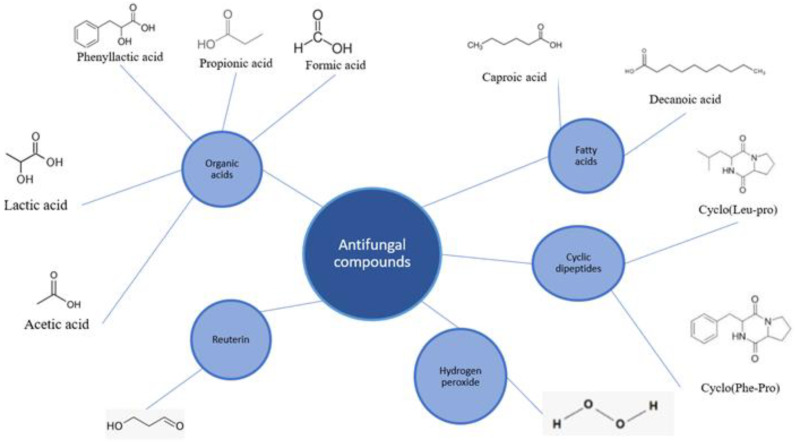
Chemical structure of some antifungal compounds produced by LABs.

LABs produce many different secondary metabolites which are not required for growth. The goal of the secondary metabolites is to change the environment in such a way that it becomes more suitable for the bacteria to live in. By inhibiting fungal growth, LABs create a more suitable environment for themselves, as they suppress the organisms that compete with them for nutrients. Several secondary metabolites have antifungal qualities. The main groups of secondary metabolites with antifungal properties are described here [61]. (see Figure 1 and Table 3)

#### 4.1.1. Organic Acids

LABs produce a variety of organic acids that can disrupt cellular metabolism thereby stopping the growth of the cells [71].

In a study to test for the antifungal activity of *Leuconostoc citreum* and *Weisella confusa* in rice cakes, the researchers found that the organic acids acetic acid and lactic acid can reduce the growth of *Cladosporium* sp.YS1, *Penicillium crustosum* YS2, and *Neurospora* sp. YS3 [72]. The researchers found that lower concentrations of acetic acid were more effective in inhibiting fungal growth than the same concentrations of lactic acid. At 35 mM, acetic acid managed to inhibit the growth of *Cladosporium* sp.YS1, *P. crustosum* YS2, and *Neurospora* sp. YS3 by 80.5, 85, and 85%, respectively. Moreover, 35 mM of lactic acid inhibited the growth of these fungi by 80, 79, and 62%, respectively.

A former study found that there is a relationship between the antifungal activity of eight different LAB species and the medium that they are grown on [73]. The different LAB strains consisted of species of *L. plantarum*, *L. paracasei*, *L. fermentum*, and *L. brevis*. The fungal species, *A. niger*, *P. roqueforti*, and *Endomyces fibuliger* were used to test the antifungal activity. When the medium was supplemented with phenyl pyruvic acid (PPA), the antifungal activity of the LABs increased. In this study, the researchers also identified a strong negative correlation between the pH and the antifungal activity, thus indicating that these acids are responsible for antifungal activity. Phenyllactic acid and its derivative hydroxyphenyllactic acid were also pinpointed as the compounds that were responsible for antifungal activity and, hence, the prevention of quick spoilage, as exerted by LABs in feed silage [74], curated meats [75], and the malting of barley [76].

#### 4.1.2. Reuterin

Reuterin is produced by several Gram-positive bacteria when they are starving [77]. Reuterin is a growth inhibitor, and it is active against a variety of organisms, namely both Gram-positive and -negative bacteria, yeast, and fungi. Among fungi, it has shown to be effective against several species, including *Fusarium* spp., *Penicillium* spp., and *Aspergillus* spp. [67]. Reuterin is produced either directly or indirectly from glycerol. LABs lack the oxidative pathway necessary for using glycerol as the sole carbon source. Thus, LABs need another carbon source to be able to degrade glycerol.

A study recently investigated the MIC of reuterin [67]. The researchers used the LAB *L. reuteri* ATCC 53608 to produce reuterin. The effects of reuterin were tested against the fungi *P. chrysogenum* LMA-212 and *Mucor racemosus* LMA-722 in yogurt. They found that, when a concentration of 5 mM or higher was reached, fungal growth was completely inhibited during an incubation period of 21 days.

Another study examined the possible synergetic effects of reuterin in combination with PLA [9]. This study used *L. reuteri* R29 to produce both reuterin and PLA. The antifungal activity was tested by the fungi *F. culmorum*, *A. niger*, and *P. expansum*. In this study, the researchers found that a medium containing 500 mM glycerol and 1.5% glucose stimulates the production of reuterin the most. When the concentration of glucose is higher, the accumulation of reuterin is inhibited by the production of 3-HPA reductase. This enzyme has a reductive effect on antifungal activity by converting reuterin to 1,3-propanediol.

#### 4.1.3. Fatty Acids

Fatty acids can also be considered antifungal compounds. They have been hypothesized to kill fungi by the disintegration of the plasma membrane [78,79]. In one study, the researchers tested whether the effectiveness of the fatty acid cis-9-Heptadecenoic acid (CHDA) was dependent on the sterol concentration present in the fungal membrane, as sterol can act as a buffer [79]. They found that the fungi *Idriella bolleyi* and *Pseudozyma rugulosa*, which are the fungi with the highest sterol content, are most resistant to CHDA. On the contrary, *Phytophthora infestans* and *Pythium aphanidermatum* oomycetes, which do not possess sterol, were the most sensitive to CHDA. CHDA is a fatty acid that is produced by the fungus *Pseudozyma flocculosa*; similar fatty acids are produced by LABs [68].

Experiments with hydroxy fatty acids’ production during sourdough fermentation showed that monohydroxy C18:1 fatty acid shows antifungal activity [80]. The fermented sourdough containing the fatty acids was then tested on bread to check for antifungal activity. In this study, the researchers used seven LAB strains that are known to convert linoleic acid. The peak of C18:1 occurs 2 days in the fermentation. Therefore, sourdough for breadmaking was fermented for two days. Then bread containing the fermented sourdoughs harboring the fermenting LABs was tested in regard to the shelf live. Compared to the control of the LABs, *L. hammesii* increased the shelf life of bread inoculated with *P. roqueforti* and *A. niger* by three and two days, respectively. It increased the shelf life of bread with environmental contaminants by seven days.

#### 4.1.4. Cyclic Dipeptides

Cyclic dipeptides are one of the most common peptide derivatives in nature [6]. Several of these cyclic dipeptides have shown antifungal activity. A study by Niku-Paavola et al. (1999) discovered new antimicrobial compounds [81]. A culture filtrate of *L. plantarum* VTT E-78076 included cyclo(glycyl-L-leucyl). Dalbello et al. (2007) found that cyclo(Phe-Pro) and cyclo(Leu-Pro) produced by *L. plantarum* FST 1.7 have antifungal activity against *F. culmorum* and *F. graminearum* [82]. Apart from the inhibition, a study found that the cyclic dipeptide cyclo(L-Leu-L-Pro) produced by *Achromobacter xylosoxidans* can also inhibit the production of the mycotoxin aflatoxin [83]. The researchers showed that the cyclic dipeptides repress the transcription of genes involved in the aflatoxin synthetic gene cluster. They inhibited the expression of the *alfR* gene. Experiments showed that enzymes necessary for the conversion of sterigmatocystin, the precursor of aflatoxin, to aflatoxin were lost leading to an inhibited production of aflatoxin. Inhibiting the production of this mycotoxin is beneficial in agriculture, as aflatoxin can be carcinogenic to humans. The literature shows that the MIC value of cyclic dipeptides is high [6,84]. However, in synergy with other antifungal compounds, they could make a valuable contribution to killing the fungal species [6].

#### 4.1.5. Hydrogen Peroxide

Hydrogen peroxide is known as a reactive oxygen species. Reactive oxygen species are dangerous to living cells, as they react with DNA, RNA, protein, and membrane lipids very easily, thereby oxidizing them. To degrade hydrogen peroxides, cells produce an enzyme named catalase [85] The fungus *Fusarium* can produce this enzyme. When using *F. graminearum* and *F. culmorum*, Ponts et al., (2009) found that, in fungi with the deoxynivalenol (DON) chemotype, the production of mycotoxin increased 2–50-fold, while for fungi with the nivalenol (NIV) chemotype, the production decreased 2–7-fold [85]. The authors showed that fungi with the NIV chemotype can handle hydrogen peroxide better, as they produce more catalase. Therefore, it was concluded that fungi under greater oxidative stress produce more mycotoxin. A more recent study shows that the antifungal activity of hydrogen peroxide is most likely low, as bacteria produced only a little [86], but that its effects is mostly in synergy with other antifungals compounds.

#### 4.1.6. Proteinaceous Compounds

Proteinaceous compounds that show antibacterial properties have been studied a lot more extensively than proteinaceous compounds that show antifungal properties [6]. Recent works in the literature show that there are some antifungal proteinaceous compounds produced by LABs that show a significant effect on the reduction of fungal growth [60,87]. In the study performed by Ma et al., (2019), the antifungal activity of different substances produced by different LAB species isolated from citrus was investigated [87]. The authors reported that when the enzyme trypsin was added to the cell-free supernatant of the LABs, the inhibitory effect of the supernatant weakened and almost disappeared.

The other study showed that *L. rhamnosus* MDC 9661 lost its antifungal activity against *M. plumbeus* and *P. aurantioviolaceum* when the culture was treated by the enzyme’s proteinase K and pepsin [60], indicating that antifungal activity is caused by proteinaceous compounds.

## 5. Mycotoxins

Plants infected by fungi will experience a decreased growth rate. Additionally, food that has been infested with fungi and, thus, has become a health hazard, will no longer be suitable for human consumption. Negative health effects occur when fungi produce mycotoxins [2,88]. Researchers identified several groups of mycotoxins produced by the fungus Fusarium: trichothecenes, mainly DON, T-2 toxin (T-2) and HT-2 toxin (HT-2); fumonisins; zearalenones (ZEN); beauvercins; enniatins; butenolide; equisetin; and fusarins. Other important mycotoxins in horticulture and food include aflatoxins (AFs), produced by *Aspergillus* species, and ochratoxin A (OTA), produced by *Penicillium* and *Aspergillus* species [89]. While investigating a widely cited claim made by the FAO that 25% of food crops were contaminated, researchers found that the prevalence of DON mycotoxins is 60% in grain, while ZEN prevalence was reported to be as high as 80% [89]. A total of 20% of samples were contaminated above the lower regulatory EU levels, while for food-grade grain samples, this percentage was below 10%. Additionally, the researchers predict that climate change will lead to a higher incidence of food contamination, making mycotoxin contamination even more dangerous in the future.

Fungi produce mycotoxins as a self-defense mechanism when they experience growth inhibition [1]. Fungi produce mycotoxins as a response to their environment. Environmental factors that can trigger mycotoxin production but have no effect on fungal growth are changes in moisture and temperature [90]. The addition of antifungal compounds can also lead to an increase in mycotoxin production, as fungi experience more stress when they come into contact with these substances. To get rid of these mycotoxins, LABs could be used. A study found that, apart from inhibiting fungal growth, LABs can also be used to detoxify mycotoxins [84]. Detoxification of mycotoxin can be performed by LABs either through degradation or mycotoxin binding.

### 5.1. Mycotoxin Degradation

In mycotoxin degradation, mycotoxins are biodegraded or transformed by microorganisms. Preferably, the biodegradation or transformation leads to detoxification [91]. The effect of LABs on the Fusarium mycotoxins DON, T-2, ZEA, and HT-2 in malting wheat grains was described in a recent study [35]. The researchers found that the LABs lessen the amount of DON, ZEA, T-2, and HT-2 by 34, 23, 58, and 73%, respectively. The researchers hypothesized that both mycotoxin degradation and mycotoxin binding contribute to the decrease in mycotoxin concentration.

The literature shows that enzymes called epoxidases play a role in the destruction of the epoxy ring in trichothecenes, which includes DON, T-2, and HT-2 [92]. Another study reports that DON detoxification via degradation can occur via 3-O-acetyltransferases encoded by the tri101 gene [93]. Therefore, the researchers conclude that degradation has an impact on the mycotoxin concentration, however, exact details on which part of the reduction is caused by degradation should be investigated in future research. Another study found that trichothecene mycotoxins can be degraded by a mixture of bacteria from the intestine of a chicken [94]. The degradation occurs via deacetylation and deep oxidation. OTA is shown to be degraded by *Pediococcus parvulus* in Douro wines. The degradation of OTA occurs via hydrolysis of the amide bond of OTA. As a result, the non-toxic products OTα and phenylalanine are formed.

Microorganisms, and more specifically LABs, can degrade mycotoxins. In the past, it proved to be difficult to find out what genes were responsible for this. Nowadays, with advancements in molecular biology and genomics, this becomes increasingly less difficult [84]. The literature shows that the *tri101 gene*, which is responsible for the 3-O-acetylation reaction, can be responsible for the acetylation of trichothecene mycotoxins, thereby detoxifying them [93,95,96,97]. A former study describes the detoxification of Zearalenones via LABs with high esterase activity [98]. Hassan et al., (2016) reported that genes such as these could be cloned in LABs to help them detoxify mycotoxins [84]. These genetically engineered LABs will be more effective in the detoxification of mycotoxin than unmodified LABs. Although the biodegradation of LABs is a permanent solution to the mycotoxin problem, it does have some problems. Some mycotoxins will be converted to even more toxic compounds, for example, the degradation from zearalenone to α-zearalenol [99]. Another study showed that AFB1 is converted to aflatoxicol [100]. In this case, the degraded molecule is less dangerous than the mycotoxin. It can, however, still generate potentially toxic effects. The degradation of mycotoxins can also be a time-consuming process [91]. For these two reasons, mycotoxin absorption is often preferred.

### 5.2. Mycotoxin Adsorption

The second way to detoxify mycotoxins is via adsorption. In adsorption, the cell wall of the bacteria binds to the mycotoxin [101]. It has been shown that thermally inactivated LABs show a higher binding capacity than active LABs [102]. This is caused by changes in the cell wall. Other factors that have been identified to affect the binding capability of LABs are the type of growth medium, state of bacteria (dead or alive), type of strain, pH of the medium, bacterial count, and incubation temperature [90].

A study suggests that 37 °C is the best temperature for the decontamination of AFB1 [103]. Fuchs et al., (2008) reported that OTA and patulin are bound optimally at a pH of 5 [104]. Adsorption of mycotoxins happens through the binding on the bacterial cell wall or via proteins present on the bacterial cell surface. The bacterial cell wall is made up of peptidoglycans. The literature shows that different mycotoxins, such as aflatoxins and fumonisin, can bind directly to peptidoglycan [105,106]. Alternatively, mycotoxins can bind to proteins present on the bacterial cell wall. Mycotoxin binding is permanent only when the bacteria die; living bacteria can release mycotoxins over time [107]. A study by Oatley et al. (2000) found that Bifidobacteria were able to adsorb 25–60% of the mycotoxin aflatoxin AFB1 [108]. A recent review showed that *L. rhamnosus* have the highest decontamination rate of AFB1, at around 80% [109]. Other researchers looked at the effectiveness. In a similar study, 20 LAB strains were tested on their binding affinity with AFB1 [101,110]. The researchers found the highest binding activity for two *L. amylovurus* strains (CSCC 5160 and CSCC 5197) and one *L. rhamnosus* strain (Lc 1/3).

### 5.3. Effect Fusarium Mycotoxins on Human and Animal Health

One of the most subtle fungi species in plant–pathogenic interactions in agriculture and horticulture is the Fusarium fungus [111]. This fungus affects crops in every climate zone around the world. The *Fusarium graminearum* and *Fusarium oxysporum* strains have even been listed in the top 10 list of fungal pathogens by a scientific paper on molecular plant pathology at places 4 and 5, respectively [112]. The list was based on their perceived importance both scientifically and economically. For this reason, the health effects of mycotoxins on humans and animals will be specified to mycotoxins produced by the fungi of the Fusarium strain. Notable mycotoxins produced by Fusarium include DON, T-2, ZEA, and fumonisin B1 (FB1) [113]. A high intake of DON can lead to abdominal distress, malaise, diarrhea, emesis, and even death in pigs [114]. A high intake of FB1, to which horses seemed to be particularly sensitive, leads to decreased cardiac output and reduced arterial pulse pressure in horses. Additionally, fumonisins can induce Equine Leuko Encephalo Malacia (ELEM) in horses. ELEM is a disease of the central nervous system that is characterized by depression, blindness, and ataxia [115].

The literature shows that both innate and adaptive immunity are affected by Fusarium mycotoxins, as they affect the production of macrophages and neutrophils, decrease the activity of t-cells and b-cells, and inhibit the production of antigens [116]. The main impact of Fusarium mycotoxins on the body of animals and humans seems to be targeted at the immune system. It is, therefore, interesting to see how a mycotoxin can aid a pathogen in the infection of a host.

A review written by Antonnissen et al., (2014) provides an excellent example of an occurrence like this [114]. The bacterium Salmonella is a well-known bacterium that can be responsible for gastroenteritis in humans and animals such as pigs and cows [117,118]. To estimate the effect of mycotoxins or mycotoxins in combination with diseases, pigs are often used, as their metabolic tracts and internal organs are very similar to those of humans [119]. A study showed that pigs experience reduced weight gain when T-2 toxin is present in their food. [120]. The researchers also discovered that the T-2 toxin reduces the motility and invasiveness of the bacterium Salmonella typhimurium. However, when the host cells are also affected by T-2, bacterial invasion may be increased.

## 6. Symbiotic Relations between Plants and LABs

The inhibition of fungal growth is not the only way in which LABs promote the growth of plants in agriculture. Bacteria and plants are known to live in symbiosis with each other. When organisms live in symbiosis, this means that they live together and provide mutual benefit to each other. Soil bacteria can aid plants in the defense against fungi, as was described in the previous chapters. Since this review is about LABs, the focus lies on the interactions between these bacteria and plants, and since LABs are used to improve plants’ growth and health, the focus also lies on the benefits that LABs can provide to plants.

A study shows that LABs, in addition to their antifungal activity, also protect plants against pathogenic bacteria. In this study, three LAB strains (KLF01, KLC02, and KPD03) were tested for their effect against a bacterium, *Xanthomonas campestris pv. Vesicatoria*, that causes spots on plants on peppers [121]. The researchers found that all LAB strains exhibit strong inhibition against the pathogenic *Xanthomonas campestris pv. Vesicatoria*, with the KLF01 stain showing the most inhibition. Additionally, the researchers wanted to test whether the LABs could also promote growth in the peppers. They did this under greenhouse conditions and field conditions. To measure growth, the researchers looked at factors such as chlorophyll production, dry weight increase, shoot length, and marketable fruit. The researchers found that, in the greenhouse, the shoot length increased significantly for the KLF01 and KPD03 strains. The root length increased the most for KLC02. Chlorophyll production went up with all LAB strains, and it went up the most for KLF01 at 15%. The plants that were treated with the LABs also exhibited a higher production yield, as more pepper fruit could be harvested per plant.

Another study looked at the effect of the plant-growth-promoting organisms *Rhodobacter sphaeroides*, *Lactobacillus plantarum*, and *Saccharomyces cerevisiae* on cucumber-plant growth [122]. The researchers show that while all organisms promote the growth of the plants, the *R. sphaeroides* promotes growth the most. The lactic acid bacteria, *L. plantarum* increased the shoot length, root length, fresh weight, and dry weight of the cucumber plants by 7%, 19%, 63%, and 40%, respectively. These numbers are lower than the numbers for the other two plant-growth-promoting organisms. The researchers also found that plants inoculated with *L. plantarum* had a significantly higher ABA production than plants inoculated with the other microorganisms and the control group. ABA is involved in stomatal closure and can be considered the stress hormone of the plant. A higher amount of ABA may be responsible for a lower growth rate of cucumbers inoculated with *L. plantarum* compared to cucumbers inoculated with the other microorganisms. Compared to the control, all microorganisms induce cucumber growth. For the lactic acid bacteria, the researchers hypothesize that this is thanks to organic acid production, which aids the plants in the uptake of nutrients, as these bacteria have the potential to solubilize phosphate and increase phosphorus nutrition [123,124].

## 7. Future Prospects

Amplified interest in sustainable food production increases the demand for natural solutions such as the usage of LABs. The substances produced by the organisms have a lower impact on human health and soil. This gives LABs huge potential in this field. However, pitfalls need to be addressed. As mentioned in the introduction, LABs have been generally regarded as safe. Nonetheless, scientific efforts in the identification of specific strains, safety evaluations, and research on health-promoting properties need to be undertaken. These features can be strain specific and, thus, need to be accurately described and categorized.

## 8. Conclusions

LAB-related research shows a lot of promise for the production of biological fungicides. The challenge remains to grow the perfect mixture of LAB strains that produce the required secondary metabolites under optimal conditions. The literature has shown that the most promising mixtures most likely contain different strains of which the chemical repertoires have an additive effect in their biocontrol of the fungus [6,61,71]. LABs additionally have the opportunity to degrade or bind toxic mycotoxins produced by the fungi. Except for antifungal activity, LABs show more ways to be beneficial for agriculture. They show benefits in other biotic stresses experienced by plants, namely against bacteria, and they can also promote the plants’ growth. When the LABs remain in the final mixture that will be added to the plants grown in agriculture, the mixture may have growth-promoting effects on the crops [122,124].

## Figures and Tables

**Table 1:** Pesticide use per continent and pesticide use in the top 5 countries [15].

Continents	Pesticide Use (tons)	Countries	Pesticide Use (tons)
Africa	82,851	China	1,763,000
Americas	1,329,563	USA	407,776
Asia	2,161,869	Brazil	377,176
Europe	478,326	Argentina	172,928
Oceania	69,725	Canada	90,839

**Table 2:** LABs that produce antifungal agents.

Name of the LAB Species	Source	Activity Spectrum	Antifungal Agents	Initial pH ^d^	Temperature ^d^	References
**Pediococcus species**						
*Pd. acidilactici*		*A. fumigatus*, *A. parasitius*, and *F. oxysporum*	A phenolic compound	6.5/6.8	20/28/37 °C	[30]
*Pd. pentosaceus*	Dairy products	*P. digitatum* and *Geotrichum candidum var citri-aurantii*	Organic acids	6	30 °C	[31]
*Pd. pentosaceus*	Dairy products	*F. graminearum*	Phenolic antioxidants	6–6.5 ^a^	37 °C	[32]
*Pd. pentosaceus HM*	Honey	*C. krusei*, *C. glabrota*, and *C. albicans*	-	5.6 ^b^	35 °C	[33]
*Pd. pentosaceus KCC-23*	Italian ryegrass	*P. chrysogenum*, *F. oxysporum*, *P. roqueforti*, *Botrytis elliptica*, and *A. fumigatus*	-	6–6.5 ^a^	30 °C	[34]
*Pd. acidilactici* and *P. pentosaceus*	CC ^e^	*F. culmorum* and *F. poae*	Organic acids	6–6.5 ^a^	32/35 °C	[35]
*Pd. acidilactici CRL 1753*	silage	*A. niger*, *A. japonicus*, *P. roqueforti*, and *Metschnikowia pulcherrima*	-	6.5	37 °C	[36]
*Pd. acidilactici*	malt	*C. albicans*	Organic acids	6–6.5 ^a^	37 °C	[37]
**Leuconostoc species**						
*L. citreum*	Italian durum wheat semolina and whole durum wheat semolina	*A. niger*, *P. roqueforti*, and *Endomyces fibuliger*	Organic acids	6–6.5 ^a^	30 °C	[38]
*L. mesenteroides*	Feta cheese and yoghurt	*P. candidum* and *Debaryomyces hansenii*	Bacteriocin	6–6.5 ^a^	30 °C	[39]
*L.* spp.	Milk bread rolls and pound cakes	*P. corylophilum*, *A. niger*, *Wallenia sebi*, and *Cladosporium sphaerospermum*	-	6–6.5 ^a^	30 °C	[40]
*L. mesenteroides*	Traditional fermented Andean food	*Meyerozyma guillermondii*, *P. roqueforti*, *A. oryzae*, and *A. niger*	Phenyllactic and 3,5-Di-O-caffeoylquinic acids	6–6.5 ^a^	30 °C	[41]
**Lactobacillus species**						
*Lacticaseibacillus. rhamnosus GR-1* and *Limosilactobacillus. reuteri RC-14*	CC ^e^	*C. glabrata*	Aggregation abilities	6–6.5 ^a^	37 °C	[42]
*Limosilactobacillus fermentum*	Cassava, a Nigerian fermented product	*A. niger*, *A. flavus*, and *P. expansum*	-	6–6.5 ^a^	37 °C	[43]
*L. helveticus*	A dairy product	*P.* sp.	Organic acids	6–6.5 ^a^	37 °C	[44]
*Lacticaseibacillus paracasei LOCK0921*	Culture collection center	*Alternari brassicicola*	-	6–6.5 ^a^	37 °C	[45]
*Latilactobacillus sakei ALI033*	Kimchi	*P. brevicompactum FIO2*	Organic acids	6–6.5 ^a^	37 °C	[46]
*Schleiferilactobacillus. harbinensis L172*	CC^e^	*P. commune*, *Galactomyces*, *Y. lipolytica*, and *Mucor racemosus*	-	4.8–4.97	10–12 °C	[47]
*Limosilactobacillus. fermentum*	Cocoa bean	*P. citrinum* and *G.moniliformis*	Organic acids	4–4.5	25 °C	[48]
*Lentilactobacillus. buchneri UTAD104*	Silage	*P. nordicum*	Organic acids	6–6.5 ^a^	30 °C	[49]
*Apilactobacillus. kunkeei*	Honeybee	*Z. rouxii*	-	6–6.5 ^a^	34 °C	[50]
*Limosilactobacillus. reuteri*	Whole wheat sourdough	*A. niger*	n-Decanoic,3-hydroxydodecanoic acid and 3-hydroxydecanoic acid	6–6.5 ^a^	37 °C	[51]
*furfurilactobacillus Rossiae*, *the companilactobacillus group*, and the *Lentilactobacillus bucheri group*	Milk bread rolls and pound cakes	*P. corylophilum*, *A. niger*, *Wallenia sebi*, and *Cladosporium sphaerospermum*	-	6–6.5 ^a^	30 °C	[40]
*the Schleiferilactobacillus perolens group*	-	*Eurotium repens*, *Wallenia sebi*, and *Cladosporium sphaerospermum*	-	6–6.5 ^a^	30 °C	[40]
*Levilactobacillus. brevis LPBB03*	coffee fruit	*A. Westerdijkiae*	-	6–6.5 ^a^	30 °C	[52]
*Limosilactobacillus fermentum*	Traditional fermented Andean products (chica and tocosh)	*Meyerozyma guillermondii*, *P. roqueforti*, *Aspergilus oryzae*, and *A. niger*	Phenyllactic and 3,5-Di-O-caffeoylquinic acids	6–6.5 ^a^	30 °C	[41]
*Lacticaseibacillus paracasei*, *Lactiplantibacillus pentosus*, *Lacticaseibacillus rhamnosus*, *Limosilactobacillus fermentum*, and *L. helveticus*	Cheese	*P. chrysogenum*, *Mucor racemosus*, *and Cladosporium harbarum*	Organic acids	6–6.5 ^a^	37 °C	[53]
*Lactiplantibacillus paraplantarum*	Fermented dates	*A. fumigates*, *Curvularia lunata*, *F. oxysporum*, *Gibberella moniliformis*, and *P. chrysogenum*	Organic acids	3	37 °C	[54]
*Lactiplantibacillus plantarum*, *companilactobacillus paralimentarius*, *Lactiplantibacillus pentosus*, *Lentilactobacillus buchneri*, and *Limosilactobacillus fermentum*	Corn silage	*F. verticilioides*	-	2.7/3.7/4.7/5.7/6.7/7.7/8.7	30 °C	[55]
*Lacticaseibacillus paracasei SYR90* and *Lacticaseibacillus rhamnosus BIOIII28*	Whey and Cheese samples	*Y. lipotica*, *R. mucilaginosa*, and *P. brevicompactum*	-	6–6.5 ^a^	30 °C	[56]
*Limosilactobacillus fermentum*, *L. sakei*, and *L. zeae*	Cheese and meat	*P. brevicompactum*	-	6–6.5 ^a^	30 °C	[56]
*Schleiferilactobacillus harbinensis K.V9.3.1 Np*	Cow milk	*Y. lipotica*	Organic acids	6–6.5 ^a^	30 °C	[57]
*Limosilactobacillus fermentum C14*	Homemade curd	*P. digitatum*, *Mucor* sp. and *Trichophyton rubrum*	Organic acids	6–6.5 ^a^	28 °C	[58]
*Lacticaseibacillus rhamnosus A238*	Biena culture collection (st-Hyacinthe, QC, Canada)	*P. chrysogenum*	Organic acids	6–6.5 ^a^	37 °C	[53]
*Latilactobacillus. sakei*	CC ^e^	*F. culmorum and F. Poae*	Organic acids	6–6.5 ^a^	30 °C	[35]
*Lactiplantibacillus pentosus LAP1*	A fermented fish product	*C. tropicalis*, *C. albicans and C. krusei*	-	3/4/5/6	30 °C	[59]
*Apilactobacillus kunkeei*	Honeybee beebread	*A. niger*, *Zygosaccharomyces rouxii*, and *Candida* sp.	-	3.0–4.0 ^c^	34 °C	[50]
*Lacticaseibacillus rhamnosus MDC 9661*	Armenian dairy product	*P. aurantioviolaceum* and *Mucor plumbeus*	proteinaceous compounds	6–6.5 ^a^	30–42 °C	[60]

*A* = *Aspergillus*, *C* = *Candida*, *F* = *Fusarium*, *P* = *penecillium*, *Pd* = *Pediococcus*, *Y* = *Yarrowia*, and *Z* = *Zygosaccharomyces*. ^a^ Based on the average MRS medium; ^b^ based on the average Sabouraud agar medium; ^c^ based on the average yeast malt agar medium; ^d^ cultivation conditions for the bacteria; ^e^ culture collection.

**Table 3:** Minimum inhibitory concentration (MIC) of several antifungal compounds based on Reference.

Compound	MIC (mM)	Activity Spectrum	References
Lactic acid	274–405	*A. flavus*	[62]
Acetic acid	38–41, 8.33, 80	*A. flavus*, *F. graminearum 623*, *A. niger*	[62,63,64]
Butyric acid	9.08	*F. graminearum 623*	[63]
Propionic acid	8.1	*F. graminearum 623*	[63]
Formic acid	19.5	*F. graminearum 623*	[63]
Caprioc acid	4.3	*F. graminearum 623*	[63]
Phenyllactic acid	45.1	*A. fumigatus* and *P. roqueforti*	[65]
cyclo(l-Phe-l-Pro)	81.9	*A. fumigatus* and *P. roqueforti*	[65]
Diacetyl	0.005	*Penicillium* spp.	[66]
Reuterin	0.1–2.0	*A. niger*, *A. versicolor*, *P. chrysogenum*, *P. citrinum*, *P. commune*, *P. crustosum*, *P. roqueforti*	[67]
decanoic acid	0.15–0.58	*P. roqueforti*, *P. commune*, *A. nidulans*, *A. fumigatus*, *P. anomala*	[68]
2-hydroxydecanoic acid	0.027–0.13	*P. roqueforti*, *P. commune*, *A. nidulans*, *A. fumigatus*, *P. anomala*	[68]
3-hydroxyundecanoic acid	0.049–0.25	*P. roqueforti*, *P. commune*, *A. nidulans*, *A. fumigatus*, *P. anomala*	[68]
Indolelactic acid	24	*P. solitum DCS 302*, *P.* sp. *nov. DCS 1541*	[69]
2-hydroxy-(4-methylthio)butanioc acid	66	*P. solitum DCS 302*, *P.* sp. *nov. DCS 1541*	[69]
2-hydroxy-3-methylbutanioc acid	42	*P. solitum DCS 302*, *P.* sp. *nov. DCS 1541*	[69]
2-hydroxy-4-methylthiopentanioc acid	38	*P. solitum DCS 302*, *P.* sp. *nov. DCS 1541*	[69]
δ-dodecalactone	1.8–3.3	*A. flavus*, *A. fumigatus*, *A. petrakii*, *A. ochraceus*, *A. nidulans*, *P. roqueforti.*	[70]
